# Temporal processing in the auditory brainstem response by full-term 6-week- and 9-month-old infants

**DOI:** 10.1038/srep12647

**Published:** 2015-07-30

**Authors:** Xiaoqin Mai, Twila Tardif, Lin Xu, Mingyan Li, Paul R. Kileny, Jie Shao, Betsy Lozoff

**Affiliations:** 1 Department of Psychology, Renmin University of China, Beijing, 100872, China; 2 Center for Human Growth and Development, University of Michigan, Ann Arbor, Michigan, 48109, USA; 3 Department of Psychology, University of Michigan, Ann Arbor, Michigan, 48109, USA; 4 Department of Child Health Care, Children’s Hospital Zhejiang University School of Medicine, Zhejiang, 310003, China; 5 Department of Otorhinolaryngology, University of Michigan, Ann Arbor, Michigan, 48109, USA; 6 Department of Pediatrics and Communicable Diseases, University of Michigan, Ann Arbor, Michigan, 48109, USA

## Abstract

Early auditory temporal processing abilities are important for language acquisition and for later reading abilities. In the present study, auditory brainstem responses (ABRs) were recorded in a forward-masking paradigm in healthy, full-term infants aged 6 weeks (n = 111) and 9 months (n = 62). Our purpose was to establish normative values of forward-masking ABRs and investigate the development of auditory temporal processing in infants at these ages. Infants were presented with pairs of stimuli (an initial “masker” followed by a “probe”) separated by different time intervals (8, 16, and 64 ms). Results showed that as masker-probe intervals became longer and as infants got older, Wave V latency to the probe shortened. The greatest improvements in Wave V latencies from 6 weeks to 9 months of age were observed in the 64-ms masker-probe interval, suggesting that central auditory nervous system related to the temporal processing at this interval might undergo rapid development during the first year of life.

The auditory brainstem response (ABR) to click stimuli can provide precise information about the functional integrity of the brainstem and other auditory structures. It has been used in hearing screening and clinical diagnosis for both audiological and neurological impairments, such as conductive and sensorineural hearing loss, cochlear lesions, tumors of the auditory nerve, and brainstem lesions^1^,^2^,^3^,^4^. The ABR components in newborns and young infants typically consist of three well-defined wave forms, Wave I (representing activity at the cochlear nerve), Wave III (activation at the cochlear nucleus), and Wave V (reflecting activity at the lateral lemniscus)^5^. Although these ABR components are similar in adults, their timing and amplitude in infants show longer latencies and less consistent amplitudes due in part to incomplete myelination of the auditory system. Thus, ABRs in young children can give information about central nervous system maturation. In particular, Wave V, which occurs roughly 6 ms after stimulus onset, is frequently used as an indicator not only of auditory processing but also the neurological integrity of the auditory system^6^.

Researchers and clinicians in a variety of fields have become increasingly interested in the function of auditory processing in subcortical pathways. In addition to simple auditory thresholds and the time it takes to perceive stimuli, the ability to process more complex auditory stimuli, and to engage in rapid auditory processing, has been found to relate to the development of language and reading skills, as well as being a potential marker for dyslexia, autism, and early hearing loss^7^,^8^. In addition to presenting complex stimuli such as music or syllables, a person’s capability to discriminate rapidly presented auditory information can be evaluated by varying click-stimulus presentation rates and through forward- or backward-masking paradigms. In these paradigms, varying the intervals between pairs of stimuli creates the potential for a “masking” effect to be created by one stimulus on another if the intervals are sufficiently small.

The forward-masking paradigm is different from simply varying the presentation rate in that there is a sufficiently long interval after the second stimulus to allow the nervous system to recover and prepare for the next pair of stimuli. Previous studies have demonstrated that increased rate of stimulus presentation or decreased masker-probe time intervals prolong Wave V latency and reduce Wave V amplitude of the ABR, particularly in young infants^9^,^10^. This phenomenon is thought to relate to delayed neural transmission, reduced synaptic efficiency, and incomplete myelination, which may also contribute to speech and language difficulties later in life^11^,^12^,^13^,^14^.

Although previous studies found that forward masking Wave V latencies were longer in newborns than adults in all examined masker-probe interval conditions (10, 50, and 100 ms), decreasing the masker-probe interval had similar effects on newborn and adult Wave V latencies^9^,^10^. We find this result to be somewhat perplexing, since developmental differences seem more plausible. We propose that the lack of significant interactions between age and masker-probe interval may be due to missing critical masker-probe intervals for observing differences and as, the authors note, small sample sizes. Allen, Miller, & DeSteno^15^ reported that many of the speech-related discriminations that are relevant to fine-tuned timing discriminations (e.g., differences between “voiced” and “unvoiced” stop consonants such as /b/, /d/, /g/ vs. /p/, /t/, /k/ in English) occur in the 50–90 ms range. We speculate that masker-probe intervals in the 50–90 ms might be more important than shorter masker-probe intervals during development. Our study further examines developmental changes in the temporal masking effect in infancy by using other masker-probe intervals and a larger sample size. Obtaining a clear understanding of the developmental trajectories involved in rapid auditory processing may have clinical significance. Researchers increasingly rely on more complex ABR responses, such as forward masking, as diagnostic indicators for language or other forms of delay in infants and young children.

The purpose of the present study was to investigate the development of temporal processing abilities in infants at two ages—6 weeks and 9 months—using a forward-masking paradigm. In addition, our study attempted to establish normative values of forward-masking ABRs for healthy, full-term infants at 6 weeks and 9 months post-partum. We focused on 6 week and 9 months infants because during the first year of the life, the human auditory system has a rapid development, and the brainstem input to the auditory cortex reaches its peak^16^. In addition, 9 months of age is the beginning of the rapid language development, which depends on efficient temporal processing of auditory information. In the present study, infants were presented with pairs of stimuli (the first stimulus is the masker and the second stimulus is the probe) separated by different time intervals (8, 16, and 64 ms). These intervals were selected based on literature on forward-masking paradigms^9^,^17^. In such paradigms, as long as the two stimuli are spaced closely in time (i.e., at 70 ms or less), both stimuli activate auditory neurons and neurons responding to the first stimulus have not recovered to become available to respond to the second stimulus. The shorter the interval between stimuli, the longer it takes for auditory neurons to recover and become available to respond^18^. Also, these intervals were doable in terms of setting up the software of the data recording device we used. We examined the effects of the interval between the stimulus pairs (masker-probe time intervals) on the response elicited by the probe (second stimulus). Since the previous research found that many of the speech-related discriminations relevant to fine-tuned timing discriminations occur in the 50–90 ms range^15^, we expected to observe developmental effects especially on the masker-probe interval at 64 ms in the present study.

## Methods

### Participants

Enrollment occurred in rural southeastern China in conjunction with a project on the brain and behavioral effects of early iron deficiency. Only healthy infants without iron deficiency anemia (IDA) were used in this developmental ABR study. All infants met the following criteria: singleton full-term birth (37–42 weeks gestation) weighing > 2,500 grams; no prenatal complications or congenital anomalies; no general undernutrition (<10th percentile for weight or length); no acute or chronic illness, no multiple or prolonged hospitalizations (>5 days). ABR recordings were obtained during sleep for 125 infants at 6 weeks and 104 infants at 9 months. The sample for analysis consisted of 111 infants aged 6 weeks and 62 infants aged 9 months (see Table 1 for the sample characteristics and family background). Because some of the 9-month-old infants awoke during testing and thus could not complete the whole protocol, resulting in different sample sizes across the three conditions (62 for the 64-ms condition, 54 for the 16-ms condition, and 49 for the 8-ms condition). In other words, among 62 infants at 9 months, 62 had ABRs for one condition (64-ms condition), 54 had ABRs for two conditions (64- and 16-ms conditions), and 49 had complete ABR data (all three conditions) recorded. Therefore, among infants at 6 weeks and 9 months, 44 had ABRs of the 64-ms condition recorded at both ages, 38 had ABRs of 64-ms and 16-ms conditions at both ages, and 34 had complete usable ABR data (all three conditions) at both ages (also see Table 2 for sample sizes in each condition).

Fourteen infants at 6 weeks were excluded from analysis for the following reasons: health exclusion (n = 3), equipment malfunction (n = 2), and one ear or both ears did not pass hearing screen (n = 9). Forty-two infants at 9 months were excluded from analysis for the following reasons: health exclusion (n = 2), did not fall asleep (n = 5), woke at the beginning of the forward-masking test (n = 3), one ear or both ears did not pass hearing screen (n = 7), artifactual data (n = 1), and IDA (n = 24). Infants with IDA at 9 months (defined as hemoglobin IDA < 110 g/L plus 2 or more abnormal iron measures) were excluded, since IDA in infancy may impair myelination, and some previous studies have found that infants with IDA have slower transmission through the auditory brainstem pathway^19^,^20^.

Informed written consent for participation in the study was obtained from the parents of all infants. Procedures were approved by the Institutional Review Boards of the University of Michigan and the Children’s Hospital of Zhejiang University and were carried out in accordance with the approved guidelines.

### ABR recording

Infants were tested during a spontaneous nap, generally while in quiet sleep. No sedatives were used. Auditory brainstem response (ABR) testing was carried out using a Biologic Navigator/Traveler (Bio-Logic Systems Corp., Mundelein, IL) evoked potential system. Responses were recorded with surface silver/silver chloride electrodes attached to the infant’s forehead using adhesive tape, in the midline below the hairline (noninverting), and to the mastoid on each side (ipsilateral as inverting and contralateral as ground electrodes, respectively). Impedance was below 10 kΩ for all recordings. While in most cases the impedance is below 5 kΩ, we used 10 kΩ in the present study because in the case of infants' oily skin it was more realistic, and we did not want to over scrub infants and wake them up. Impedance bellow 10 kΩ is common in electrophysiological studies with special populations, such as children, schizophrenics, and other populatoins^21^,^22^.

At least two independently averaged waveforms were recorded in response to each stimulus. Each averaged response consisted of 1,300 accepted sweeps. If the Wave V of the two waveforms were comparable, the two waveforms would be added later to yield a waveform representing 2600 responses. If the two 1300 waveforms were not replicable, an additional one was recorded until replicate waveforms were observed. The data acquisition program automatically rejected any traces contaminated by high-amplitude artifacts (voltage exceeding ± 23.80 μV), and the electroencephalogram was amplified and band-pass filtered from 30 to 1,500 Hz. It should be noted that we used 30 Hz instead of 100 Hz high pass filter because it helps enhance Wave V and the post V negativity SN 10, which makes the identification of wave V easier. At 100 Hz we cut off much of the frequency range that contains wave V especially in infants. In the pediatric literature 30 Hz is more common. Some studies show the advantage of a lower than 100 Hz high pass filter setting^23^,^24^. Also, all of the automated ABR screening devices use 30 Hz or lower for their detection algorithm.

**Figure 1:**
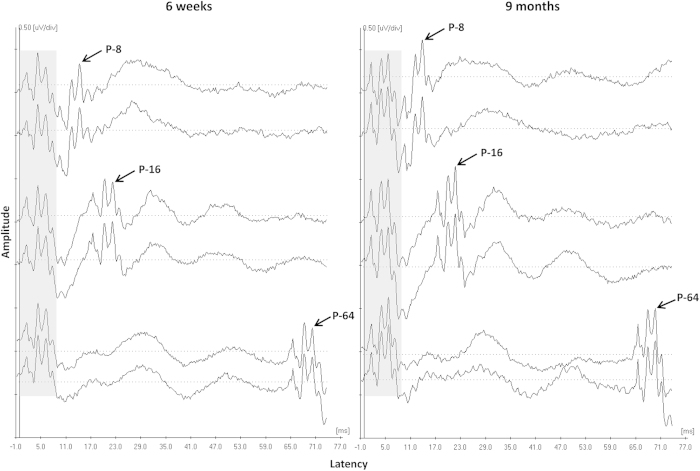
Auditory brainstem responses for three masker-probe conditions are shown for one participant at 6 weeks (left) and 9 months (right). To show the reproducibility of ABR waves, two traces are presented for each condition: 8-ms (top two traces), 16-ms (middle two traces), and 64-ms conditions (bottom two traces). The waves evoked by the first stimulus (masker) are highlighted in gray areas. Wave V evoked by the second stimulus (probe) is denoted by the arrow. It should be noted that ABRs were time-locked to the masker, and thus the latency of Wave V evoked by the probe was calculated by subtracting the masker-probe interval, i.e., 8, 16, and 64 in corresponding conditions, from the latency value of Wave V showed in the figure.

Infants were initially presented with 0.1 ms click stimuli at 30 dB nHL as hearing screen. If a replicable wave V response was obtained, testing was concluded with the infant passing the hearing screen on a given ear. Infants who passed the hearing screen in both ears continued to the forward-masking paradigm, in which an initial 0.1 ms click stimulus (the masker) was presented at 80 dB nHL, followed by an identical stimulus (the probe). The time between the masker and probe was varied at intervals of 8, 16, and 64 ms. The masker-probe pairs were delivered to each ear by means of insert transducers at a rate of 11.7/sec. The recording epoch was 74.67 ms. Figure 1 presents examples of the waveforms of ABRs for three conditions: 8-ms (top two traces), 16-ms (middle two traces), and 64-ms interval (bottom two traces).

### ABR scoring

The ABR data were analyzed off-line. The Biologic program read the stored data and displayed the averaged responses on the computer screen. For each condition (8, 16 and 64 ms), the replicated waveforms were added, yielding a waveform representing 2600 responses, which was used in data analysis. With the aid of cursors, the Wave V peaks for each condition were identified and marked by a trained technician and were confirmed by a second person. The peaks were labeled without awareness of age group. Wave V latency for the probe (the second stimulus) response was identified as the peak latency. Per usual practice, the latency values obtained for the right and left ears were averaged so that each infant was represented by one value (averaged over a total of 5200 sweeps per condition) in any given group mean.

### Statistical analyses

All statistical analyses were performed using SPSS 18 (SPSS Inc., Chicago, IL, USA). Descriptive statistics provided means and standard deviations of latencies for Wave V in different conditions at the two ages. Pearson’s correlations were calculated to examine the relation between ABRs at 6 weeks and 9 months. To investigate ABR differences as a function of the masker-probe interval, a repeated measures analysis of variance (ANOVA) was conducted for Wave V latency at 6 weeks and 9 months, respectively, with experimental condition (masker-probe intervals of 8, 16, and 64 ms) as a within-subjects factor.

### Results

Table 2 shows means and standard deviations (SD) of Wave V latency in the different conditions at 6 weeks and 9 months, as well as Pearson correlation coefficients between Wave V measures at 6 weeks and 9 months. These values could be used as normative for 6-week and 9-month-old infants using this forward-masking paradigm. As expected, the 9-month ABR data were positively correlated with the 6-week ABR data in each condition (*p*s < 0.001), suggesting that the development of auditory temporal processing abilities is stable during the first year of life.

To investigate ABR differences as a function of the masker-probe interval, repeated measures ANOVAs of ABRs depending on masker-probe intervals was conducted and results showed the expected condition effect on Wave V latency at 6 weeks (*F*_(2,220)_ = 119.83, *p* < 0.001) and 9 months (*F*_(2,96)_ = 166.00, *p* < 0.001). At both ages, pairwise comparisons with Bonferroni corrections showed that the latency shortened as the masker-probe interval increased (*p*s < 0.001).

**Figure 2:**
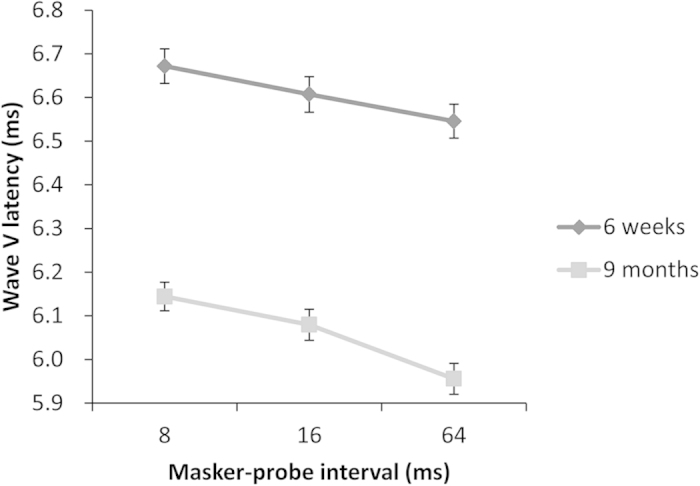
The interaction in Wave V latency between the masker-probe interval and age (n = 34).

For the 34 infants with complete data at both 6 weeks and 9 months, repeated measures ANOVAs were conducted for Wave V latency with age (6 weeks and 9 months) and experimental condition (masker-probe intervals of 8, 16, and 64 ms) as two within-subject factors. For Wave V latency, there were significant main effects of age (*F*_(1,33)_ = 546.72, *p* < 0.001) and condition (*F*_(2,66)_ = 134.82, *p* < 0.001), as well as an interaction between them (*F*_(2,66)_ = 10.30, *p* < 0.001). Specifically, latency shortened with both increasing age and increasing masker-probe interval. Moreover, the decrease in latency between 6 weeks and 9 months was greater in the 64-ms condition than in the 8- or 16-ms conditions (see Fig. 2). The mean differences were 0.528 ms, 0.528 ms, and 0.590 ms in 8-, 16-, and 64-ms conditions, respectively.

### Discussion

Our study offers a set of values that can be used as normative for infants at 6 weeks and 9 months using this forward-masking paradigm. At both 6 weeks and 9 months, Wave V latency was prolonged for the shorter masker-probe intervals. These results replicate previous studies on forward-masking effects on ABRs using noise burst maskers and click probes with newborns and adults^9^,^10^,^25^,^26^ and toneburst maskers and probes with adults^17^,^27^. Wave V latency shortened increased in all three forward-masking conditions between 6 weeks and 9 months. More importantly, we found the interaction in Wave V latency between the masker-probe interval and age such that the greatest change in Wave V latency was observed in the 64-ms condition.

Establishing normative values for forward-masking ABRs in infancy are important for this reason. Quite a bit is known about how simple ABRs vary with age. It is essential to understand how and whether age affects complex ABRs, such as ABRs of the forward-masking paradigm, and to develop normative data so that these methods might ultimately be used clinically for individual diagnosis, rather than only for research studies across groups of infants and children with particular characteristics. There is still a long way to go from research to clinical application, of course. However, the data from the present study might provide a useful reference for further research regarding the early diagnosis of relatively subtle temporal processing difficulties in infants that could lead to language and reading difficulties later in life.

The few other studies using the forward-masking paradigm in infancy have not been able to investigate developmental changes in the temporal masking effect in infancy. The pioneering study of Lasky and Rupert^10^ examined the temporal masking effect in 12 newborns and 12 adults with a click stimulus following a 50 ms broad-band masker, with an masker-probe interval of 10, 50, or 100 ms. In that study, as in ours, Wave V latency was prolonged as the masker-probe interval decreased, and this was true for both newborns and adults. However, they did not find an interaction between age and masker-probe interval in Wave V latencies. This is inconsistent with other studies which investigated auditory temporal processing by varying stimulus presentation rates and found that increasing the rate of stimulus presentation had greater effects on ABRs in infants younger than three months than in adults^28^,^29^. The authors attributed the lack of different patterns in newborns and adults to two factors: (1) the small number of masker-probe intervals, which may have inadvertently missed a critical masker-probe interval for observing differences; and (2) the small sample size. In the present study, the greatest developmental effects were observed in the longest masker-probe interval (64-ms condition), suggesting that this interval is important for observing the improvement of temporal processing abilities in the developing infant’s central auditory nervous system.

In the first year of life, the central auditory nervous system undergoes profound changes, including rapid increase in axonal myelination, dendritic arborization, and synaptic organization in brainstem pathways, which might result in the improvement of forward-masking processing. In particular, the greatest improvement seems to appear at the longest forward-masking interval during the first year of life, indicating that the auditory nervous system related to the temporal processing at this interval (64 ms) might undergo faster development during this period, compared to that at shorter intervals (8 and 16 ms). We thus speculate that the development of temporal processing capacity would have a trend of from the largest forward-masking interval to the smallest one. Future studies with more age groups are needed to confirm or refute this possibility.

There is another possible explanation. The greatest improvements in Wave V latencies from 6 weeks to 9 months of age appearing in the 64-ms forward-masking interval might have adaptive significance for language development during this period. Since phonetic discrimination requires rapid auditory processing, the increase in native-language phonetic perception between 8 and 10 months of age might relate to the improvement of temporal processing abilities during this time period^30^. Most importantly, many of the speech-related discriminations that are relevant to fine-tuned timing discriminations occur in the 50-90 ms range. Individual differences in voice onset timing are up to 40 ms, and contextual variations produce variations in an individual speaker’s productions of up to 30–40 ms^15^. Thus, over-sensitivity to small variations could lead to difficulty in categorizing within a phoneme, whereas under-sensitivity to the 60–70 ms range could lead to difficulty in distinguishing between voiced vs. unvoiced consonants. It is therefore both interesting and important to consider that the maximal sensitivity to forward-masking intervals at the 9-month age period was observed in the 64 ms condition, a range that is essential for phoneme categorization and speech perception, more generally.

The maximal developmental effect from 6 weeks to 9 months was observed at the 64-ms masker-probe interval, which is within the range (50–90 ms) of many of the speech-related discriminations. However, to validate the previous findings of this important range, it is important to include additional intervals, such as 50 ms and 90 ms, in future studies. In addition, future studies including the adult group are needed to address whether this developmental effect would preserve in adults.

The key finding in the present study may have important implications for infant development and assessment. The 64-ms forward-masking interval appears to be a particularly sensitive interval during the first year of life. In addition, the present study provides normative values for a forward-masking ABR paradigm for full-term infants at 6 weeks and 9 months. These findings may provide a useful reference for further research regarding the early diagnosis of relatively subtle temporal processing difficulties in infants that could lead to language and reading difficulties later in life^11^,^31^.

## Additional Information

**How to cite this article**: Mai, X. *et al*. Temporal processing in the auditory brainstem response by full-term 6-week- and 9-month-old infants. *Sci. Rep*. **5**, 12647; doi: 10.1038/srep12647 (2015).

## Figures and Tables

**Table 1:** Infant characteristics and family background^1^.

	6-week ABR sample	9-month ABR sample
	(*n* = 111)	(*n* = 62)
Sample charateristics		
Gender (% male, *n)*	46%, *51*	45%, 28
Birth weight (kg)	3.40 (0.43)	3.47 (0.42)
Gestational age (d)^2^	276.92 (7.30)	276.44 (7.75)
Chronological age (d)	43.18 (2.17)	279.99 (7.61)
Weight (kg)	5.07 (0.60)	9.32 (1.20)
Length (cm)	56.5 (2.3)	72.5 (2.5)
Head circumference (cm)	38.2 (1.0)	44.5 (1.4)
Family background		
Mother education (y)	11.3 (2.8)	11.0 (2.6)
Father education (y)	11.6 (2.8)	11.2 (2.7)
Residence (% rural, *n*)	69%, *76*	*73%, 45*

^1^Continuous variables are represented as mean (SD). Dichotomous variables (gender and living area) are shown as percentage and number.

^2^The gestational age was determined by the first day of the last menstrual period.

**Table 2:** Descriptive statistics of Wave V latency in different conditions at 6 weeks and 9 months.

masker-probe intervals	6 weeks			9 months			Correlation 6 wk to 9 mo	
	N	mean	SD	N	mean	SD	N	*r*
8 ms	111	6.65	0.21	49	6.12	0.20	34	0.74*
16 ms	111	6.60	0.22	54	6.06	0.21	38	0.81*
64 ms	111	6.54	0.21	62	5.96	0.23	44	0.76*

*p < 0.001.
